# The SARS-CoV-2 Lambda variant and its neutralisation efficiency following vaccination with Comirnaty, Israel, April to June 2021

**DOI:** 10.2807/1560-7917.ES.2021.26.45.2100974

**Published:** 2021-11-11

**Authors:** Neta Zuckerman, Ital Nemet, Limor Kliker, Nofar Atari, Yaniv Lustig, Efrat Bucris, Dana Bar Ilan, Miranda Geva, Reut Sorek-Abramovich, Chen Weiner, Nir Rainy, Adina Bar-Chaim, Patricia Benveniste-Levkovitz, Ramzia Abu Hamed, Gili Regev-Yochay, Ofra Hevkin, Orna Mor, Sharon Alroy-Preis, Ella Mendelson, Michal Mandelboim

**Affiliations:** 1 Central Virology Laboratory, Public Health Services, Ministry of Health and Sheba Medical Center, Tel-Hashomer, Israel; 2 Sackler Faculty of Medicine, Tel-Aviv University, Tel-Aviv, Israel; 3 Shamir Medical Center, Be'er Ya'akov, Israel; 4 Sheba Medical Center, Tel-Hashomer, Israel; 5 Public Health Services, Ministry of Health, Jerusalem, Israel

**Keywords:** COVID-19, BNT162b2 vaccination, neutralizing, variants, C.37 (Lambda Variants)

## Abstract

The SARS-CoV-2 Lambda (Pango lineage designation C.37) variant of interest, initially identified in Peru, has spread to additional countries. First detected in Israel in April 2021 following importations from Argentina and several European countries, the Lambda variant infected 18 individuals belonging to two main transmission chains without further spread. Micro-neutralisation assays following Comirnaty (BNT162b2 mRNA, BioNTech-Pfizer) vaccination demonstrated a significant 1.6-fold reduction in neutralising titres compared with the wild type virus, suggesting increased susceptibility of vaccinated individuals to infection.

The severe acute respiratory syndrome coronavirus-2 (SARS-CoV-2) Lambda variant of interest (VOI) (Phylogenetic Assignment of Named Global Outbreak (Pango) lineage designation C.37) was first detected in Lima, Peru in August 2020 and, by April 2021, the proportion reached nearly 100% of sequenced genomic isolates detected in Peru [1]. Its spread in South America, and specifically its progression to predominance in Peru, occurred despite the presence of additional lineages, including SARS-CoV-2 variants of concern (VOC) Alpha (Pango lineage designation B.1.1.7) and Gamma (Pango lineage designation P.1), suggesting high transmissibility of this variant [1-3]. However, information on Lambda resistance to the Comirnaty (BNT162b2 mRNA, BioNTech-Pfizer, Mainz, Germany/New York, United States (US)) vaccine against coronavirus disease (COVID-19) compared with other strains is currently limited [4]. Herein, we describe the appearance of the Lambda variant in Israel in April–June 2021 and the neutralising response of sera from 36 naive individuals following two vaccination doses of Comirnaty, against the Lambda variant.

## The Lambda variant in Israel

**Figure 1:**
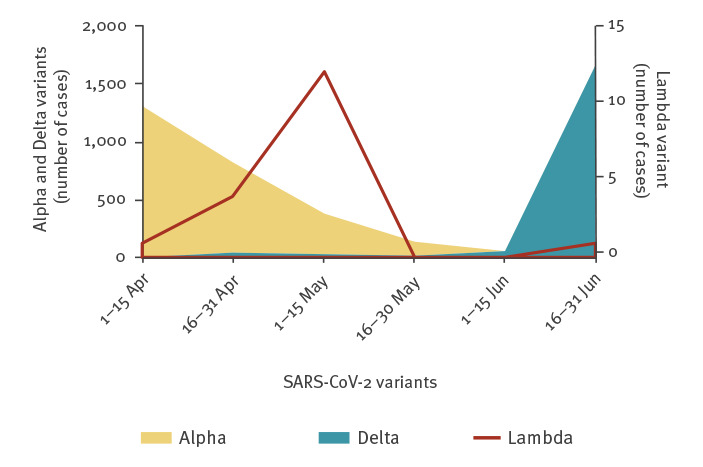
Numbers of the SARS-CoV-2 Lambda, Alpha and Delta variants in Israel, April–June 2021 (n = 4,453)

A national consortium of SARS-CoV-2 sequencing was established in Israel in December 2020, dedicated to identifying circulating and imported variants. Using these surveillance data, the first Lambda variant imported to Israel was detected on 4 April 2021, carried by travellers arriving from Argentina; this occurrence was followed by additional importations from Spain, Portugal and France. As of 31 June 2021, 18 individuals were confirmed by sequencing to be infected with the Lambda variant. Five out of the 18 individuals were vaccinated with two doses of the Comirnaty vaccine (Supplementary Table S1). The Lambda variant did not spread further within the country; during this period, the Alpha VOC was the dominant strain from February until the end of May 2021 until it was outcompeted by the Delta (Pango lineage designation B.1.617.2) VOC (Figure 1).

SARS-CoV-2: severe acute respiratory syndrome coronavirus 2.

Number of COVID-19 cases with the Lambda variant (red) in Israel between 1 April–31 June 2021. One case was observed between April 1–15, four cases between April 16–31, 12 cases between May 1–15 and one case between June 16–31. At the beginning of June, the Alpha variant (yellow) decreased to less than 50 cases, whereas the Delta variant (blue) rapidly increased to 1,655 cases by the end of June.

**Figure 2:**
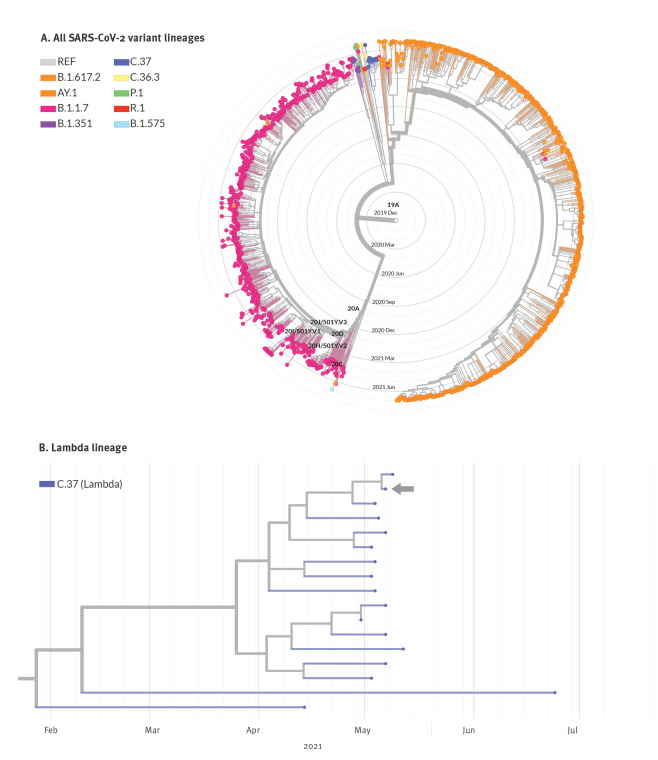
Phylogenetic tree of SARS-CoV-2 variant lineages in Israel, April–June 2021 (n = 1,380)

A phylogenetic tree of imported and circulating SARS-CoV-2 lineages in Israel in May–April 2021, constructed via the Nextstrain Augur pipeline [5], shows the dominating Alpha and Delta variants in Israel (Figure 2A). Epidemiological investigations of 18 COVID-19 cases caused by the Lambda variant with sequencing identified two main connected transmission chains and additional isolated incidents (Figure 2B).

REF: reference strain. SARS-CoV-2: severe acute respiratory syndrome coronavirus 2.

A. A phylogenetic tree representing randomly sequenced samples from COVID-19 cases in Israel between April and June 2021 (n = 1,380). Variants are indicated in colours and include Lambda (Pango lineage designation C.37; n = 17), Alpha (Pango lineage designation B.1.1.7; n = 600), Delta (Pango lineage designation B.1.617.2 and AY.1; n = 750), Beta (Pango lineage designation B.1.351; n = 6), C.36.3 (n = 4), P.1 (n = 4), R.1 (n = 2) and B.1.575 (n = 1). The phylogenetic tree was constructed via Nextstrain Augur pipeline [5].

B. The SARS-CoV-2 Lambda samples in the phylogenetic tree (n = 17), including two isolated incidents and the two transmission chains. Accession numbers (see Supplementary Table S1) for the Lambda sequences are deposited in GISAID (https://www.gisaid.org). One of the 18 total Lambda cases identified with sequencing was not included in the tree due to poor genomic coverage. The arrow represents the Lambda isolate used in the neutralisation assays (hCoV-19/Israel/CVL-13489-ngs/2020). The phylogenetic tree was constructed via Nextstrain Augur pipeline [5].

## Comirnaty vaccine effectiveness against the Lambda variant

The extent of neutralisation of the Lambda variant was examined in serum samples taken from 36 individuals (mean age: 49 years, range: 27–66; 14 men, 22 women) vaccinated with two doses of Comirnaty, 1 month following the second vaccination. The neutralising titres of the Lambda variant were compared with the wild type (WT) SARS-CoV-2 and the Alpha and Delta VOC. Cultured virus samples from SARS-CoV-2-infected individuals were characterised using the following isolates: the WT sub-lineage B.1.1.50 (hCoV19/Israel/CVL-45526-ngs/2020), Alpha (hCoV-19/Israel/CVL-46879-ngs/2020), Delta (hCoV-19/Israel/CVL-12804/2021) and Lambda (hCoV-19/Israel/CVL-13489-ngs/2020) via whole genome sequencing (Illumina COVID-seq kit on Illumina NovaSeq, Cambridge, United Kingdom). We added inactivated sera double-diluted from 1:8 to 1:16,384 to virus suspensions containing a median tissue culture infectious dose of 100 TCID_50_ of each SARS-CoV-2 variant or the WT isolate in 96-well plates for 60 min at 33°C. Virus–serum mixtures were then added to Vero-E6 cells and incubated for 5 days at 33°C, after which Gentian violet staining (1%) was used to stain and fix the cell culture layer to investigate the cytopathic effect (Supplementary methods). A dilution equal to 1:10 or above was considered neutralising.

**Figure 3:**
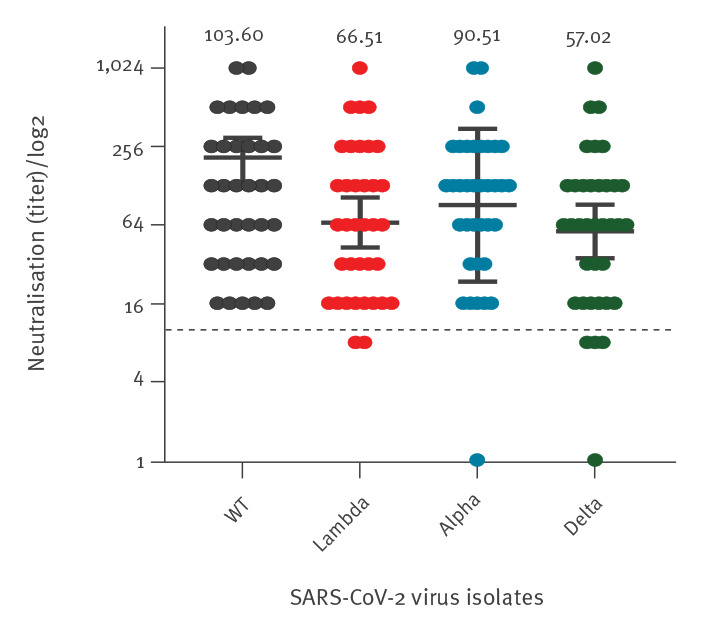
Neutralisation capacity against the SARS-CoV-2 Lambda variant using sera from Comirnaty-vaccinated individuals, Israel, April–June 2021 (n = 36)

The geometric mean titre (GMT) was 103.6 (95% confidence interval (CI): 67.6–158.6) against the WT virus, 66.5 (95% CI: 42.8–103.4) against Lambda, 90.5 (95% CI: 57.3–143) against Alpha and 57 (95% CI: 35.5–91.6) against Delta (Figure 3; Supplementary Figure S1). Wilcoxon matched-pairs signed-rank test demonstrated significant differences in neutralising titres between the WT virus and the Lambda (1.6-fold decrease, p < 0.0001) and Delta (1.8-fold decrease, p < 0.0001) VOC, but not with the Alpha VOC (1.1-fold decrease, not significant), similar to previous observations [6].

SARS-CoV-2: severe acute respiratory syndrome coronavirus 2.

Neutralisation assays were carried out with Vero-E6 cells infected with the SARS-CoV-2 Lambda, Alpha and Delta variants in addition to a WT strain using sera from 36 individuals obtained at least 1 month following the second dose of the Comirnaty (BNT162b2 mRNA, BioNTech-Pfizer, Mainz, Germany/New York, United States) vaccine. Bars represent the log geometric mean titre (GMT) and 95% confidence intervals. The value above each bar represents the geometric mean titre (GMT) of each isolate. A dilution equal to 1:10 or above was considered neutralising (dashed line).

### Ethical statement

The protocol was approved by the Institutional review board of the Sheba Medical Center (#7045-20-SMC). Written informed consent was obtained from all participants.

## Discussion

The SARS-CoV-2 virus is responsible for over 200 million cases of COVID-19 and 4 million deaths worldwide since December 2019. As the pandemic has progressed, SARS-CoV-2 variants have emerged; those with increased transmissibility are classified by the World Health Organization (WHO) as VOC and VOI [7]. The SARS-CoV-2 Lambda variant, currently classified as a VOI at the time of writing, has spread to at least 30 countries worldwide, including numerous European countries, with highest numbers in South America [1-3]. The Lambda genome is characterised by numerous amino acid substitutions and deletions in the Spike protein, including G75V, T76I, Δ246–252, L452Q, F490S, D614G and T859N [1]. The variant was first imported into Israel in April 2021 from Argentina, followed by additional importations from Europe until June 2021.

The Comirnaty vaccine, approved by the US Food and Drug Administration in December 2020, has shown to be 95% effective in preventing symptomatic COVID-19 and to provide protection against the WT SARS-CoV-2 virus and the Alpha VOC [8-10]. To date, the SARS-CoV-2 Lambda variant has not spread further within Israel, which may be attributed to the time of importation of this variant. By April 2021, the rate of vaccination in the Israeli population was high, ranging from 70% in the beginning of April to 80% by the end of June [11], leading to a decrease in the circulation of the Alpha variant, which was the dominant variant in Israel since February 2021. As a result of the effective vaccination campaign and restrictions on travel into Israel, the number of daily diagnosed individuals decreased to under 100 in mid-May [11]. The paucity in infected individuals at the time facilitated sequencing of viral samples from most SARS-CoV-2-positive individuals, leading to better identification of imported variants and transmission chain breakage. Among the 18 Lambda-infected individuals identified in Israel, five were fully vaccinated with Comirnaty, four of whom were under 50 years of age (Supplementary Table S1).

A reduction in neutralisation capacity following vaccination with Comirnaty was reported against the Alpha and Delta variants [6,12,13], as well as against other VOC such as Beta (Pango lineage designation B.1.351), Gamma and Mu (Pango lineage designation B.1.621) [12,14-16]. However, despite observed reduction in neutralisation against these latter variants, they did not become globally widespread and some were replaced by infiltration of more transmissible variants, such as the Delta variant [17]. The reasons for this can include higher infectiveness or transmissibility of some VOC compared with others, as was recently suggested for the Delta variant [18].

Limitations of the study include the small number of sera analysed and the lack of T-cell response evaluation.

In this study, neutralisation assays of the Lambda VOI with sera from 36 individuals vaccinated with two doses of the Comirnaty vaccine demonstrated a subtle yet statistically significant 1.6-fold decrease in neutralisation capacity of the Lambda variant compared with the WT SARS-CoV-2 strain. Using the same cohort, neutralisation capacity of the Delta variant showed a significant reduction of 1.8-fold, while the Alpha variant had a 1.1-fold decrease compared with the WT strain, similar to previous reports [6,12,13].

## Conclusions

The vaccine efficacy against the Lambda VOI was similarly compromised as compared with the Delta VOC. We speculate that infection of vaccinated individuals with the Lambda variant may be explained by the gradual reduction in neutralising antibodies in time. Overall, data presented herein contribute to the growing evidence of Comirnaty vaccine efficacy against known VOI/VOC and emphasise the importance of vaccination together with public health-related restrictions in efforts to control virus spread.

## Supplementary Data

453000 Supplement
