# Supplementary material for: The SARS-CoV-2 Lambda variant and its neutralisation efficiency following vaccination with Comirnaty, Israel, April to June 2021
Source: Euro Surveill. 2021 Nov 11;26(45):2100974. doi: 10.2807/1560-7917.ES.2021.26.45.2100974 (PMC8646983; doi:10.2807/1560-7917.ES.2021.26.45.2100974)

Supplementary Appendix

This supplementary material is hosted by *Eurosurveillance* as supporting information alongside the article "Prevalence of the Lambda (C.37) variant and its neutralization efficiency following BNT162b2 vaccination" on behalf of the authors who remain responsible for the accuracy and appropriateness of the content. The same standards for ethics, copyright, attributions and permissions as for the article apply. Supplements are not edited by *Eurosurveillance* and the journal is not responsible for the maintenance of any links or email addresses provided therein.

Methods

Ethical statement

The protocol was approved by the Institutional review board of the Sheba Medical Center. Written informed consent was obtained from all participants.

Viral isolation

Using sequencing we identified 3 nasopharyngeal samples from SARS-CoV-2 positive individuals which contained the WT sub lineage B.1.1.50 (hCoV19/Israel/CVL-45526-ngs/2020), B.1.1.7 (Alpha, hCoV-19/Israel/CVL-46879-ngs/2020), B.1.617.2 (Delta, hCoV-19/Israel/CVL-12804/2021) and C.37 (Lambda, hCoV-19/Israel/CVL-13489-ngs/2020). Confluent VERO-E6 cells were incubated for 1 hour at 33°C with 300 µl of nasopharyngeal samples containing the viruses followed by addition of 5 ml 2% FCS MEM-EAGLE medium. Upon CPE detection, supernatants were aliquoted and stored at -80°C.
In order to calibrate and determine the 50% endpoint titer (TCID50) of each variant VERO-E6 cells at concentration of 20*103/well were seeded in three sterile 96-wells plates with 10% FCS MEM-EAGLE medium, and stored at 37°C for 24 hours. Ten-fold serial dilutions of each variant were prepared using 2% FCS MEM-EAGLE medium and incubated for five days with the VERO-E6 cells. Following Gentian Violet staining TCID50 of each variant was calculated using the Spearman-Karber method.

SARS-CoV-2 micro-neutralization assay

VERO-E6 cells at concentration of 20*103/well were seeded in sterile 96-wells plates with 10% FCS MEM-EAGLE medium, and stored at 37°C for 24 hours. One hundred TCID50 of WT, Alpha, Delta and Lambda SARS-CoV-2 isolates were incubated with inactivated sera diluted 1:10 to 1:1280 in 96 well plates for 60 minutes at 33ºC. Virus serum mixtures were added to the Vero E-6 cells and incubated for five days at 33ºC after which Gentain violet staining (1%) was used to stain and fix the cell culture layer. Neutralizing dilution of each serum sample was determined by identifying the well with 4 of the highest serum dilutions without observable cytopathic effect. A dilution equal to 1:10 or above was considered neutralizing.

Statistical Methods

Before-after plots of log-transformed neutralizing antibodies and Geometric Mean Titers (GMT) with confidence interval (CI) of 95% were performed using GraphPad Prism 5.0 (GraphPad Software, Inc., San Diego, CA).

Supplementary Table 1. Lambda variant cases imported into Israel between April and June 2021. The table includes data per each patient infected with the Lambda variant in Israel – age, gender, country of importation ("-" denotes no known importation), vaccination status and known association to a transmission chain or an isolated event without known transmission (transmission/isolated), where "?" denotes unknown. Sequences were deposited to GISAID (https://www.gisaid.org/) and their accession numbers are provided, with the exception of one sample which had a poor coverage.

Date infected	age	gender	country of importation	vaccinated	transmission/ isolated	GISAID accession #
2021-04-30	45	F	-	yes	transmission	EPI_ISL_2183528
2021-05-03	4	M	Spain	no	transmission	EPI_ISL_2183589
2021-05-03	16	F	Portugal	no	transmission	EPI_ISL_2183598
2021-05-03	45	F	Portugal	no	transmission	EPI_ISL_2183602
2021-04-30	6	M	Spain	no	transmission	EPI_ISL_2183618
2021-04-30	40	M	-	no	transmission	EPI_ISL_2183619
2021-05-05	22	M	Spain	no	?	EPI_ISL_2183683
2021-05-07	15	F	-	no	transmission	EPI_ISL_2183708
2021-05-07	47	M	Portugal	no	transmission	EPI_ISL_2183709
2021-05-07	13	F	Portugal	no	transmission	EPI_ISL_2183713
2021-05-07	3	F	Portugal	no	transmission	EPI_ISL_2183715
2021-05-07	10	F	Portugal	no	transmission	EPI_ISL_2183718
2021-04-14	29	M	Argentina	yes	isolated	EPI_ISL_6161272
2021-04-26	38	F	Spain	no	transmission	EPI_ISL_6161559
2021-05-04	61	M	Spain	no	?	NA
2021-05-09	50	F	-	yes	?	EPI_ISL_6161677
2021-05-12	85	M	-	yes	?	EPI_ISL_6161798
2021-06-24	32	F	France	yes	isolated	EPI_ISL_6161886

Supplementary Figure 1. Neutralization capacity against the Lambda variant, paired representations. Neutralization assays were carried out with VERO-E6 cells infected with the Lambda (A), Alpha (B) and Delta (C) variants compared to a WT strain using sera from 36 individuals obtained at least 1 month following the second dose of the corminaty BNT162b2 vaccine. A dilution equal to 1:10 or above was considered neutralizing (dashed line).

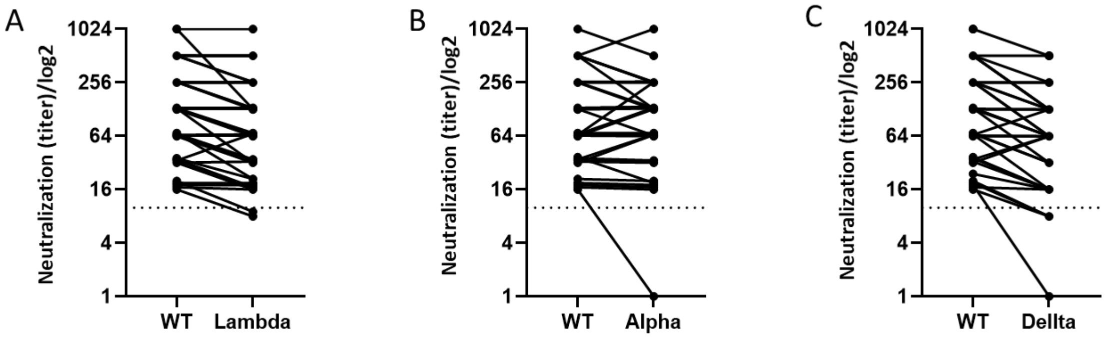

Supplement: Supplement [file 21-00974_MANDELBOIM_Supplement.pdf]
